# Endophytic *Beauveria bassiana* Induces Oxidative Stress and Enhances the Growth of *Fusarium oxysporum*-Infected Tomato Plants

**DOI:** 10.3390/plants11223182

**Published:** 2022-11-21

**Authors:** Felix Nchu, Neo Macuphe, Ilyaas Rhoda, Lee-Ann Niekerk, Gerhard Basson, Marshall Keyster, Ninon G. E. R. Etsassala

**Affiliations:** 1 Department of Horticultural Sciences, Faculty of Applied Sciences, Cape Peninsula University of Technology, P.O. Box 1905, Bellville 7535, South Africa; 2 Environmental Biotechnology Laboratory, Department of Biotechnology, University of the Western Cape, Private Bag X 17, Bellville 7535, South Africa

**Keywords:** endophytic *Beauveria bassiana*, *Fusarium oxysporum*, tomatoes, antioxidant activities, oxidative stress

## Abstract

Studying the mechanisms through which endophytic fungi confer protection to host plants against parasites will contribute toward elucidating the endophytic fungi–plant–pathogen relationship. In this study, we evaluated the effects of endophytic *Beauveria bassiana* on the antioxidant activity, oxidative stress, and growth of tomatoes infected with the fusarium wilt pathogen, *Fusarium oxysporum* f. sp. lycopersici (FOL). Tomato seedlings were inoculated with *B. bassiana* conidia and then contaminated with FOL experimentally. Four treatments (Control [T1], FOL only [T2], *B. bassiana* only [T3], and *B. bassiana* and FOL [T4]) were assessed. The plants from the *B. bassiana* and FOL treatment (T4) were significantly taller (DF = 3, 56; *p* < 0.001) and produced more leaves and aerial part biomass than those treated with only FOL (T2). Remarkably, plants in the two treatments with FOL (T2 and T4) had the lowest antioxidant activities; meanwhile, plants from the FOL treatment (T2) had the lowest ROS (superoxide and hydroxyl radicals) contents. Broadly, strong positive correlations between ROS and all the plant growth parameters were recorded in this study. While the current results revealed that the endophytic entomopathogen *B. bassiana* enhanced antioxidant capacity in plants, it did not improve the antioxidant capacity of *F. oxysporum*-infected plants. It is possible that the pathogenic FOL employed a hiding strategy to evade the host immune response and the antagonistic actions of endophytic *B. bassiana*. In conclusion, *B. bassiana* inoculum enhanced the growth of tomatoes infected with FOL, induced higher oxidative stress in both *F. oxysporum*-infected and -uninfected tomatoes, and improved antioxidant activities in plants inoculated with *B. bassiana* only.

## 1. Introduction

*Fusarium oxysporum* is a causative agent of the destructive vascular wilt disease in tomatoes. The pathogen infects the roots and then spreads to the vasculature [1,2]. Vascular wilt disease is responsible for huge crop production losses [3]. Pathogenic *F. oxysporum* strains are difficult to control. Its conidia can remain dormant in soil and plant tissues for many years during unfavourable conditions [4]. The pathogen begins its infective cycle as a biotroph; over time, as the infection progresses, it changes to a necrotroph [1]. Current control measures include crop rotation, synthetic fungicides, and planting resistant tomato varieties [2]. However, synthetic fungicides are costly and can induce pathogen resistance to fungicides and contaminate the environment. These setbacks have intensified the search for biorational control approaches.

After many years of research, endophytic microorganisms have immerged as promising alternative control agents against many destructive phytopathogens in crop production [5]. Fungal endophytes can decrease pathogen virulence and disease severity [6]. Endophytic fungi have many attractive attributes that make them potential candidates for developing profitable and eco-friendly antifungal products. Endophytic fungi can colonize and form a symbiotic relationship with plant hosts without causing infection [7]. During symbiosis, endophytes protect host plants against pathogens while depending on the host plants for nutrients and habitat [8]. Fungal endophytes can confer protection to host plants through direct or indirect pathogen antagonism [9]. Direct antagonistic effects include direct competition with and parasitism on phytopathogens and antibiosis through the secretion of enzymes and toxins with direct adverse effects on phytopathogens [10]. Indirect antagonistic effects involve eliciting and enhancing host resistance through the expression of defence-eliciting genes in host plants—potentially reducing oxidative stress in host plants [7], improving the production of secondary metabolites [11], and promoting plant growth through phytohormone-mediated induction and antioxidant activities [12,13].

However, despite the progress made toward understanding and breaking down the complex endophyte–plant–pathogen systems, many essential knowledge gaps still exist. While many studies have examined the tripartite endophyte–plant–pathogen relationship, the underlying mechanism of how disease-modifying endophytes interact with plants and pathogens is unclear [14]. Elucidating the underlying mechanism by which endophytes reduce pathogen virulence and plant disease severity is essential to facilitate endophytic fungi’s transformation into viable, effective biopesticides [15].

According to Card et al., 2016 [7], indirect antagonistic effects appear to be the more plausible mechanism by which endophytes confer protection to plants, and they proposed that further studies on oxidative stress and antioxidant activities will provide a better understanding of the interaction between fungal endophytes, plant hosts and phytopathogens. It was suggested by Bacon and White and White and Torres [16,17] that endophytes can induce plant production of antioxidants by producing reactive oxygen species; the antioxidants will then protect symbiotic plants from pathogen and environmental stresses. Furthermore, White, 2010 [17] proposed the need to evaluate the hypothesis that antioxidants are responsible for enhanced stress tolerance in endophyte-infected plants. The triggering of localised cell death in the host plants by fungal endophytes has been put forward as another strategy used by endophytes to protect plants against phytopathogens [2,7]. The roles of oxidative stress and antioxidant activities during pathogen colonisation need to be clarified.

In this study, two well-known fungal species, *Beauveria bassiana* and *Fusarium oxysporum* f. sp. lycopersici (FOL), and tomato (*Solanum lycopersicum*) were used to study the influence of endophytic fungi on oxidative stress and antioxidant activities in an endophyte–plant–pathogen relationship. We hypothesised that endophytic *B. bassiana* could improve the growth of *F. oxysporum*-infected tomatoes by enhancing the antioxidant activities of tomatoes and suppressing oxidative stress. *B. bassiana* is an ideal fungus because it has fascinating traits relevant to pest control [18]. It can colonise many plant species endophytically, naturally or experimentally; it can cause natural epizootics among insect populations; it can be cultured on artificial media easily; it is a facultative endophyte; it is ubiquitous and soil-borne; it can induce plant growth, it can enhance tissue nutrient contents and secondary metabolite production in plants, and it is capable of phytopathogen antagonism [7,18,19,20,21]. *F. oxysporum* is a root-infecting hemibiotrophic fungus that can cause destructive vascular wilt disease in tomatoes [1,22]. During infection, *F. oxysporum* produces toxins, including fusaric acid, and hijacks many phytohormone pathways in the host to facilitate disease progression [3]. These toxins may induce oxidative stress and elicit antioxidant activities in plants. 

Recently, Rojas et al. [23] reported that isolates of *Anthracocystis flocculosa* and *Penicillium olsonii* significantly reduced *Fusarium* head blight symptoms in wheat when they were applied two or three days before the pathogen inoculation; however, the authors did not determine whether the activity of the fungi was due to direct antibiosis, or indirect via competition of plant-mediated defence activation. Combès et al. [24] found that the endophyte *Paraconiothyrium variabile* has a vigorous antagonistic activity against *F. oxysporum* using optic and electronic microscopies, and they attributed its actions to competition-induced metabolite production. De Lamo [2] argued that the protection of tomatoes by endophytic *F. oxysporum* from disease severity by pathogens appears to be distinct from systemic resistance (SR) or systemic acquired resistance (SAR). In this study, we focused on reactive oxidative stress and antioxidant activities to better understand the relationship and the mechanisms by which endophytes confer protection to tomatoes against a pathogenic strain of FOL (UPFC). The objectives of this study were to determine the effects of endophytic *B. bassiana* on the antioxidant activity, oxidative stress, and growth of tomatoes infected with a fusarium wilt pathogen, FOL.

## 2. Results

### 2.1. Characterization of the FOL

**Figure 1:**
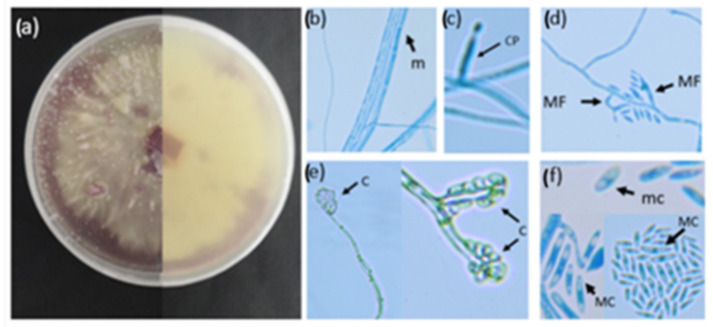
Micromorphological characteristics of FOL (Isolate UPFC): (**a**) Colony morphology on PDA front (left) and back of the plate (right), (**b**) aseptate mycelia—m, (**c**) initial formation of conidiophore—CP, (**d**) Monophialides—MF, (**e**) formation of head of conidia—C, and (**f**) oval shaped microconidia—mc and sickle-shaped macroconidia—MC.

The morphological and molecular characterization of the *F. oxysporum* strain (UPFC) confirmed that the fungal isolate is FOL. Based on the micromorphological characteristics, the fungal strain was identified as *Fusarium oxysporum* f. sp. lycopersici (Figure 1). The closest match of the sequence following a BLASTn search was *Fusarium oxysporum* f. sp. lycopersici 4287, accession number (CM000590.1), percentage ITS identity (99%), and FOL strain F41 translation elongation factor 1-alpha (EF1a) gene (99% identity match). Also, a mock-inoculation with the FOL strain did not cause disease in green peas (Green arrow), soya beans (SPR048-100), and sweetcorn (Golden Bantam). 

### 2.2. Growth Parameters

FOL negatively affected plant growth parameters (height and leaf number). Generally, inoculation of the tomato plants with *B. bassiana* inoculum alleviated the negative effect of the FOL strain on plant growth. The plants that were inoculated with *B. bassiana* and FOL (T4) were significantly (DF = 3, 56; *p* < 0.001) taller and (DF = 3; χ^2^ = 13.14; *p* < 0.01) produced more leaves than the plants that were treated with *F. oxysporum* only (Table 1).

### 2.3. Dry Weight and Fresh Weight

**Figure 2:**
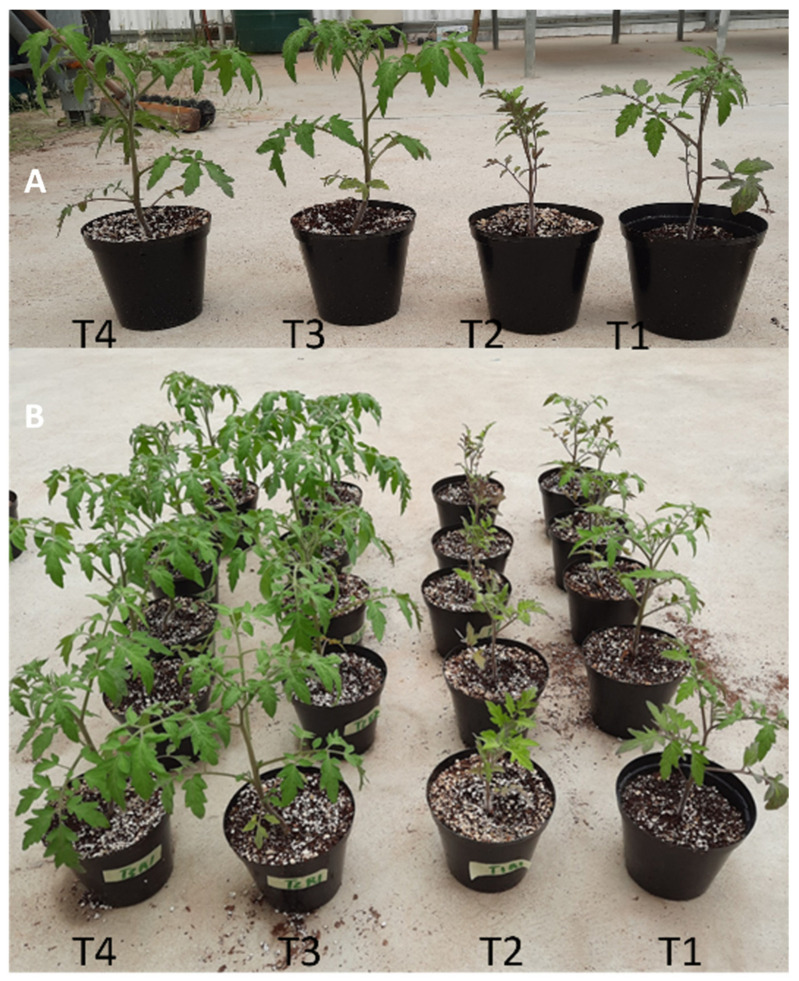
Effect of endophytic *B. bassiana* inoculum on the growth of *F. oxysporum*-infected tomato plants: T4 (*B. bassiana* and FOL), T3 (*B. bassiana*), T2 (FOL) and T1 (Control) treatments: effect on plant heights (**A**) and leaf discoloration in T2 (**B**).

*F. oxysporum* negatively affected plant growth parameters (fresh and dry weights), as shown in Figure 2. The tomato plants from treatment T4 had significantly higher fresh and dry weights of the aerial and root parts compared to the control plants and the plants treated with FOL only (T2) (DF = 3, 56; *p* < 0.001). However, the fresh and dry weights of plants from the *B. bassiana* and FOL treatment (T4) were not significantly different from plants exposed to only *B. bassiana* (T3) (Table 2).

### 2.4. Antioxidant Activities

Antioxidant activities varied significantly among the treatments, with *B. bassiana*-inoculated tomato plants (T3) yielding the highest antioxidant activity in the TEAC bioassay. Inoculating plants with FOL (T2) was associated with lower antioxidant activities. Based on the FRAP bioassay, plants inoculated with both *B. bassiana* and FOL (T4) produced a significantly (*p* < 0.05) lower mean antioxidant activity than the control-treated plants. A similar trend occurred in the TEAC results. Plants inoculated only with *B. bassiana* (T3), and those in the control (T1) treatment yielded significantly higher antioxidant activity than those in *B. bassiana* and FOL treatment (T4) (Table 3); *B. bassiana* did not improve the antioxidant capacity of FOL-infected plants.

### 2.5. Reactive Oxygen Species Assays

#### 2.5.1. Cell Death

While more cell death occurred in plants inoculated with only FOL (T2), no significant difference was observed among the treatments (DF = 3, 8; F = 1.15; *p* = 0.3) (Table 4).

#### 2.5.2. Superoxide

Generally, there was a significant difference in superoxide radical concentrations among the treatments (DF = 3, 8; F = 2.20; *p* = 0.001). Interestingly, plants inoculated with both *B. bassiana* and FOL (T4) or *B. bassiana* only (T3) yielded significantly higher superoxide levels. In contrast, the plants inoculated with FOL (T2) had the lowest concentration of superoxide in the aerial parts (Table 5). It appears *B. bassiana* inoculation induced higher superoxide and antioxidant concentrations in tomato plants compared with the phytopathogen FOL, which induced lower superoxide and lowered antioxidant levels in tomatoes. However, weak correlations between superoxide radical species and antioxidant activities were observed in this study—TEAC (y = −4.0156x + 857.87; R^2^ = 0.276) and FRAP (y = −2.9696x + 547.52; R^2^ = 0.4369). Interestingly, hydroxyl radical content had strong positive correlations with the plant growth parameters; R^2^ ranged from 0.73 to 0.98.

#### 2.5.3. Hydroxyl Radical (^•^OH)

Generally, there was a significant difference among the treatments (DF = 3, 8; F = 4.13; *p* = 0.04) (Table 6). Interestingly, plants inoculated with both *B. bassiana* and FOL (T4) had a higher hydroxyl radical concentration than those inoculated with only FOL (T2). Also, *B. bassiana* inoculation (T3) induced a significantly higher hydroxyl radical level in tomato plants than FOL inoculation (T2). Weak negative correlations between hydroxyl radical content and antioxidant activities in the different treatments were obtained in this study—TEAC (y = −1.0138x + 765.61; R^2^ = 0.0279) and FRAP (y = −0.8918x + 494.82; R^2^ = 0.0625). FOL inoculation (T2) induced the lowest oxidative stress level compared to the other treatments, including the control treatment. Remarkably, hydroxyl radical content showed strong positive correlations with the plant growth parameters; R^2^ ranged from 0.65 to 0.98.

## 3. Discussion

This study was premised on the hypothesis that endophytic *B. Bassiana* would increase plant growth and antioxidant activities and reduce oxidative stress in response to *F. oxysporum* infection. Plants inoculated with both *B. bassiana* and FOL (T4) showed enhanced growth in all parameters assessed compared with the control and FOL treatments. Literature abounds with empirical evidence of plant growth and defence-promoting properties of endophytic fungi [25,26]. Based on the current plant growth results, it can be argued that the endophytic *B. bassiana* strain (SM3) used in this study protected tomato plants against FOL infection. The higher plant heights obtained among plants exposed to *B. bassiana* might be due to the growth-promoting and nutrient uptake properties of *B. bassiana* [21] and antioxidant activities [27]. These properties could be described as indirect antagonistic effects against *F. oxysporum* infection [11]. Card et al. (2016) [7] classified the antagonistic mechanisms of an endophyte toward a pathogen into four main categories—antibiosis, competition, host-induced resistance, and direct parasitism—and further argued that indirect antagonisms, such as antibiosis and host-induced resistance, are the most probable mechanisms by which endophytic fungi confer protection to plants against well-known plant pathogens. Interestingly, although *B. bassiana* did not improve the antioxidant capacity of extracts of *F. oxysporum*-infected plants, the highest growth, considering all plant growth parameters measured, was observed in the *B. bassiana* and FOL treatment (T4), suggesting that mechanisms other than the antioxidant mechanism are essential in determining the positive plant-growth influence of *B. bassiana* inoculation.

While the current results revealed that the endophytic entomopathogen *B. bassiana* enhanced antioxidant capacity in plants, it did not improve the antioxidant capacity of *F. oxysporum*-infected plants. In this study, the enhancement of antioxidant activities by *B. bassiana* occurred in plants inoculated with *B. bassiana* inoculum only. Remarkably, we found weak negative correlations between oxidative stress factors (superoxide and hydroxyl radical) and the antioxidant activities of plants in the different treatments. Understanding the physiological implications of these findings in relation to conferring protection to plants against phytopathogen infections is crucial. Anjum et al. [28] suggested that higher antioxidant capacity is associated with better plant growth because of their ability to scavenge free radicals that may cause damage to plants. Earlier, Card et al. [7] postulated that endophytes could stimulate a defence response by inducing oxidative stress and cell death in host plants. Our results corroborate this assertion since tomatoes inoculated with *B. bassiana* inoculum had higher ROS contents and plant growth. It, therefore, makes sense to insinuate that the strong correlations between high ROS contents and plant growth obtained in this study could be linked to separate yet multiple indirect antagonistic mechanisms produced by endophytic *B. bassiana* in colonised plants, and these include induced oxidative stress, production of growth hormones and tissue nutrient-uptake. The cell’s redox state affects its proliferation/differentiation program, and controlled oxidation is essential in the early stages of the plant cell cycle [27]. Future studies should investigate the involvement of the growth-promoting and tissue nutrient-uptake properties of *B. bassiana*, as well as the relationship between oxidative stress and growth hormones, to throw more light on the endophyte–plant–pathogen relationship.

Entomopathogenic fungi are rich in compounds with antioxidant activities [29], and high antioxidant capacity could help in scavenging excessively produced ROS during infection. So why did the *B. bassiana* inoculum not induce higher antioxidant activities in *F. oxysporum*-infected tomatoes? One of the best-known responses of plants to the presence of microbial pathogens is the production of a high concentration of ROS, also known as oxidative burst. To evade a high concentration of ROS, invading pathogens employ antioxidant enzymes as the first line of defence [30]. According to Singh et al. [31], phytopathogens employ countermeasures consisting of intricate mechanisms for ROS perception, ROS neutralisation, and protection from ROS-mediated damage. However, in the current study, the inoculation of tomatoes with FOL neither induced high levels of ROS nor antioxidant capacity. Even more puzzling, the pathogen seemed capable of suppressing the ability of the endophyte (*B. bassiana*) to generate higher antioxidant capacity in the infected plants—low ROS and antioxidant activities were recorded in plants infected with FOL. Perhaps the pathogen evades recognition by the host and endophyte by deploying a hiding strategy; for example, fungal pathogens can modulate the amount of certain cell wall components to avoid recognition by host immune cells [32]. The mechanisms of ROS modulation in endophyte–plant–pathogen interactions need to be further researched to fully decipher the link between high endophyte-induced ROS content and plant growth.

## 4. Materials and Methods

### 4.1. Research Design

A greenhouse experiment was carried out for this study at the Cape Peninsula University of Technology (CPUT), Bellville campus, Western Cape, South Africa. A *Beauveria bassiana* (SM3) strain was used against the FOL strain (UPFC). In the greenhouse study, potted tomato plants were exposed to four treatments in a completely random design with a single factor. Treatment one (T1) plants were not exposed to fungus (control), treatment two (T2) plants were exposed to FOL only, treatment three (T3) plants were exposed to *B. bassiana* only, and treatment four (T4) plants were exposed to *B. bassiana*, first, and then FOL. The effect of *B. bassiana* on growth, antioxidants and oxidative stress was assessed. 

### 4.2. Plant Materials

Tomato (*S. lycopersicum* L.) seedlings (cultivar: Floradade) were purchased from Stodels Nurseries (Pty) Ltd. in Bellville, Western Cape Province, South Africa. They were maintained at the Cape Peninsula University of Technology glasshouse nursery at the Bellville Campus, Western Cape, South Africa. The tomato seedlings were kept under the following conditions: 28 ± 2 °C, 60–80% RH, and a 14/10 natural light/dark regime. Each tomato plant was gently removed from a six-pack seedling tray and transplanted into a substrate mix with a 1:1:1:1 ratio of silica sand, peat moss, vermiculite, and perlite. Before transplanting, the medium was sterilised with 1% sodium hypochlorite for 15 min and rinsed with sterile distilled water three times. The plants were fed using recommended commercial hydroponics fertiliser Nutrifeed^®^ hydroponic fertiliser (Starke Ayres Pty. Ltd., Cape Town, South Africa). The fertiliser was mixed at a recommended concentration of 10 g/5000 mL, and each potted tomato plant was drenched with 200 mL once a week. Furthermore, each tomato plant was watered with distilled water once a week for six weeks.

### 4.3. Fungus Preparation

#### 4.3.1. *Beauveria bassiana*

An existing *B. bassiana* (SM3) strain was previously isolated from a vineyard and identified molecularly [20,21]. The following method was used to culture the fungus. Briefly, the *B. bassiana* strain was cultured on a selective medium consisting of half-strength (19.5 g/1000 mL) potato dextrose agar (PDA) (Sigma-Aldrich Pty. Ltd., Johannesburg, South Africa), 0.04 g streptomycin, and 0.02 g ampicillin sodium salt. The PDA was prepared on 9 cm diameter Petri dishes, and fungal cultures were incubated as described in the Macuphe et al. (2021) method [21]. The matured conidia were harvested using a sterile spatula and transferred into a 50 mL centrifuge tube containing 25 mL sterile water. The centrifuge tube was capped and shaken for 3 min and mixed vigorously for 2 min using a vortex mixer (MI0101002D Vortex Mixer, Silverton Machines, Inc., East Longmeadow, MA, USA) at 3000 rpm to homogenise the conidial suspension. Furthermore, the homogenous conidial suspension was transferred into 1000 mL bottles comprising 500 mL sterile distilled water and 0.05% Tween 80 (polysorbate, Sigma-Aldrich, Johannesburg, South Africa). The conidia concentration was determined using a hemocytometer (Bright-Line, Sigma-Aldrich, Johannesburg, South Africa) and observed with a light microscope at 400× magnification to determine the required concentration of (1 × 10^8^ conidia mL^−1^). Germination percentage was assessed as described by Latifian and Rad (2012) [33] on a 100-spore count at 40× magnification. Each plate was replicated four times, and over 90% germination was observed.

#### 4.3.2. *Fusarium oxysporum*

Clean fungal cultures of a strain of *F. oxysporum*, a pathogen causing fusarium wilt disease in tomatoes presently being maintained in the Department of Horticulture Research Laboratory, CPUT, were used for this experiment [34]. However, it was necessary to ascertain that the fungal *F. oxysporum* strain used in this study was indeed FOL using standard morphological and molecular identification methods, since the fungal strain had not been characterised before.

##### Micromorphological Characterisation of FOL

Spores were harvested when 5-day-old fungal colonies were drenched in sterile water and dislodged from the PDA (Potato dextrose agar, Sigma-Aldrich) medium using a sterile spatula. The mycelial suspension was then strained through cheesecloth to obtain a spore suspension. The micromorphological characteristics, such as the presence or absence of septation, spores and conidiophores, were visualised using a modified version of the slide culture technique by Johnson (1946) [35]. A sterile microscope slide was placed in a petri dish containing moist filter paper. Thereafter, a 1 cm^2^ section of PDA was transferred to the microscope slide, and 10 µL of a spore suspension was added to the centre of the PDA square. A cover slip was placed on top of the PDA, and the petri dish was sealed with parafilm. The plates were incubated on a benchtop at room temperature for 2 weeks until mycelial growth was visible. Following the incubation, the coverslip was removed and placed on a clean microscope slide containing lactophenol blue staining solution (Lactophenol cotton blue, Sigma-Aldrich) and viewed under the microscope (Zeiss PrimoStar, 40×) (Carl Zeiss (Pty) Ltd., Cape Town, South Africa). All images were captured using a Canon 80D digital camera (lens: Canon EF-S 10–18 mmf/4.5–5.6 IS STM).

##### Molecular Characterisation of FOL

Approximately 100 mg of fungal biomass was collected from a PDA plate. The total genomic DNA was extracted using the Zymo Research Quick-DNA Fungal/Bacterial Miniprep Kit (Zymo Research, catalogue number D6005). The purity and concentration of the DNA were measured using a NanoDrop™ 2000 spectrophotometer (Thermo Scientific). A fraction (100 ng) of the genomic DNA was used to classify the fungi using PCR markers (ITS1 and ITS4) and the translation elongation factor 1-alpha (TEF1 or EF1-α) gene for the phylogenetic analysis [36,37]. The PCR amplicons were sequenced on an ABI3730xl Genetic Analyser at the Central Analytical Facility (CAF, Stellenbosch University, Stellenbosch, South Africa) using the ITS1 primer and Fa+7 (AACGTCGTCGTCATCGGCCACGTCGACTCT). The DNA sequence was submitted to BLASTn (https://www.yeastgenome.org/blast-fungal; accessed on 7 November 2022) to identify potential hits for the isolates [38].

##### Pathogenicity of the FOL Strain against Other Heirloom Crop Cultivars

The roots of one-week-old seedlings of some heirloom crop cultivars (supplied by Seeds for Africa, Pty Ltd., Cape Town, South Africa), were mock-inoculated, and the symptoms of plant disease were observed. Five seedlings of green peas (Green arrow), soya beans (SPR048-100), and sweetcorn (Golden Bantam) were individually submerged in a FOL conidial suspension at a concentration of 1 × 10^6^ conidia mL^−1^ for 24 h. Control plants were not exposed to the fungus. The plants were transplanted into pots containing a substrate mix of equal parts of sphagnum peat moss, perlite, and compost. The substrate mix was drenched with the hydroponics fertiliser (Nutrifeed®) (Starke Ayres Pty Ltd., Cape Town, South Africa). Plants were checked regularly for symptoms associated with FOL over three weeks.

### 4.4. Greenhouse Experiment

This experiment was done at the CPUT glasshouse nursery, Bellville campus, in the Department of Horticultural Sciences, Western Cape, South Africa. The glasshouse nursery had the following conditions: 25 ± 5 °C, 65% ± 5% relative humidity, and the average light intensity was 31.77 kilolux. Two-week-old tomato seedlings were transferred into 15 cm pots containing a substrate mix of silica sand, peat moss, vermiculite, and perlite in a ratio of 1:1:1:1. Twenty plants were placed into 15 cm pots separately. This experiment had four treatments: T1 = control, T2 = FOL, T3 = *Beauveria bassiana* and T4 = *Beauveria bassiana* with *Fusarium oxysporum*. The conidial suspensions comprised of conidia FOL or *B. bassiana* and 0.05% Tween 20 prepared at 1 × 10^8^ conidia mL^−1^ were used to inoculate plants. Each plant was drenched with 100 mL of conidial suspension, and each treatment had five plants representing five replicates (n = 20). However, the control plants were drenched with 100 mL of sterile distilled water (0.05% Tween 20). The plants were fed using a recommended commercial hydroponics fertiliser Nutrifeed^®^ (Starke Ayres Pty. Ltd., Cape Town, South Africa) that had the following ingredients: N (65 mg kg^−1^), *p* (27 mg kg^−1^), K (130 mg kg^−1^), Ca (70 mg kg^−1^), Cu (20 mg kg^−1^), Mo (10 mg kg^−1^), Fe (1500 mg kg^−1^), Mg (22 mg kg^−1^), S (75 mg kg^−1^), B (240 mg kg^−1^), Mn (240 mg kg^−1^), and Zn (240 mg kg^−1^). The fertiliser was concentrated with sterile distilled water to 10 g/5000 mL, and 200 mL was added to each tomato plant weekly. Moreover, each tomato plant was watered twice a week with distilled water. The collected data were plant height, the number of leaves, and fresh and dry weights of aerial and root parts. Plant height was measured from the soil surface to the top of the highest leaf; the number of leaves was counted in each plant. Furthermore, aerial parts were separated from the roots and weighed (g plant^−1^). Tomato plants were dried at room temperature (25 °C) for 21 days, and dried plants were weighed (g plant^−1^). The experiment was repeated three times.

#### Re-Isolation of Fungi from Tomato Leaves

**Figure 3:**
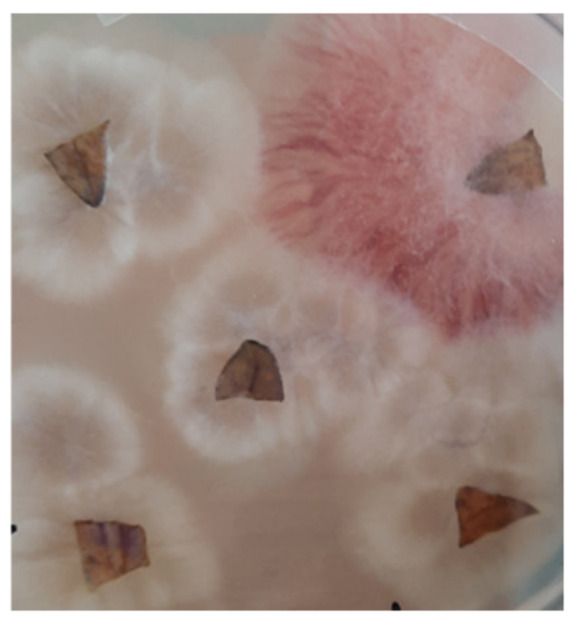
Fungal (*B. bassana* and *F. oxysporum* f. sp. lycopersici) outgrowths from leaf sections.

The colonisation of the tomato leaves by *B. bassiana* and FOL at 21 days was measured by re-isolation following surface sterilisation. Three rectangular leaf sections with veins (2 mm^2^) were carefully excised from an older leaf from each plant and transferred to the laboratory on ice. The leaf sections were individually surface-sterilized with 0.5% sodium hypochlorite for 1 min, followed by 70% ethanol for 1 min and rinsed twice in sterile distilled water and placed on the selective medium (19.5 g potato dextrose agar [PDA], 0.02 g/L of ampicillin [Sigma-Aldrich], and 0.04 g/L streptomycin [Sigma-Aldrich, Johannesburg, South Africa]). The leaf sections were visually examined daily for the presence of any fungal growth. Conidia and hyphae were also microscopically examined to identify the fungi [35,39,40]. *B. bassiana* was successfully re-isolated from at least one leaf section of plants exposed to T3 and T4 treatments, and FOL was re-isolated from at least one leaf section from T2 and T4 treatments (Figure 3). No fungus was re-isolated from the control plants. The presence of fungal mycelial outgrowth in at least one of the leaf sections was considered an indication of the successful colonisation of a plant. 

### 4.5. Antioxidants

#### 4.5.1. Sample Preparation

The dried samples were ground, and the powdered material was transferred into plastic bags. Five tomato samples per treatment were randomly selected from the three repetitions and weighed (n = 20), and 0.1 g of powdered tomato samples was transferred into 50 mL centrifuge tubes. Tomato samples were extracted with 25 mL of 60% ethanol and placed inside the incubator for 24 h [21].

#### 4.5.2. Ferric Reducing Antioxidant Power (FRAP)

The ferric-reducing antioxidant power assay method by Benzie and Strain (1996) [41] was used in this study. The assay is based on reducing the ferric-tripyridyltriazine compound to ferrous in the presence of antioxidants. The reagent was used as follows: 2.5 mL of a 10 mmol/L TPTZ (2,4,6-tripyridyl-s-triazine, Sigma-Aldrich, Johannesburg, South Africa) solution in 40 mmol/L HCl plus 2.5 mL of 20 mmol/L FeCl_3_ and 25 mL of 0.3 mol/L acetate buffer and kept at pH 3.6 was prepared freshly and warmed at 37 °C [21]. The aliquots of 40 μL of the sample supernatant were mixed with 0.2 mL sterile distilled water and 1.8 mL FRAP reagent. Following the incubation at 37 °C for 10 min, the spectrophotometric method was used to determine the absorbance of the reaction mixture at 593 nm. The standard solution was 1 mmol/L FeSO_4_, and the result was captured as the concentration of antioxidants with a ferric reducing capacity of 1 mmol/L FeSO_4_.

#### 4.5.3. Trolox Equivalent Antioxidant Capacity (TEAC)

The TEAC method was used to assess the antioxidants’ potential to scavenge free radicals in tomatoes in a method described by Miller et al. (1993) [42]. The TEAC value is calculated by comparing the antioxidant’s ability to scavenge the blue-green coloured 2,2′-azino-bis-(3-ethylbenzothiazoline-6-sulphonic acid) ABTS^•+^ radical cation to the water-soluble vitamin E analogue’s ABTS^•+^ radical cation scavenging ability.

### 4.6. Oxidative Stress

#### 4.6.1. Cell Viability Assay Using Evan’s Blue Solution

A method described by Gokul et al. [42] was employed to determine the cell viability of tomato leaves. 5 × 1 cm^2^ leave sections were excised from the leaves of each leaf stage. The excised leaf sections were placed into 1.5 mL Eppendorf tubes containing 1 mL of 0.25% (*w*/*v*) Evan’s Blue solution. Samples were incubated at room temperature (21 °C) for an hour in Evan’s Blue solution. After the incubation period, unbound Evan’s Blue solution was rinsed off, and samples were incubated in deionised water for 12 h at room temperature (21 °C). After the 12-h incubation period, deionised water was decanted and replaced with 1 mL of 1% (*w*/*v*) sodium dodecyl sulfate (SDS) solution. The leaf blocks were crushed in SDS solution with a small pestle and placed on a heating block for an hour at 65 °C. Samples were centrifuged at 13,000 rpm for 5 min. This was conducted to pellet plant material. The supernatant was loaded onto a 96-well microtitre plate and read at 600 nm on a spectrophotometer [42]. Three tomato samples per treatment were randomly selected from the three repetitions.

#### 4.6.2. Superoxide Determination

A method by Gokul et al. [43] was used to determine the superoxide radical concentration in tomato plants from the different treatments. Eight 1 cm^2^ sections excised from the leaves of each leaf stage were placed in 800 µL of potassium phosphate buffer (pH 7.0) [50 mM potassium phosphate buffer (pH 7.0) containing 10 mM potassium cyanide, 10 mM hydrogen peroxide, 2% SDS and 80 µM NBT]. Leaf materials were incubated in this buffer for 20 min at 21 °C and crushed within the solution with a miniature pestle. The samples were centrifuged at 13,000 rpm for 5 min, and the supernatant was transferred to a clean 2 mL Eppendorf tube. A volume of 200 µL of the sample was loaded onto a microtitre plate and read at 600 nm on a spectrophotometer. The calculation was conducted to determine the superoxide radical using the extinction coefficient 12.8 cm^−1^ [43]. Three tomato samples per treatment were randomly selected from the three repetitions.

#### 4.6.3. Hydroxyl Radical Determination

A method adapted from Halliwell et al. [44] was employed to determine and analyse the hydroxyl radical content within tomato trifoliate. Fifty milligrams (50 mg) of frozen ground-up leaf material was used and placed into separate 1.5 mL Eppendorf tubes. To these tubes, 1 mL of 10 mM phosphate buffer (pH 7.4) [containing 15 mM 2-Deoxy-d-Ribose] was added. The tubes containing the samples were then incubated for 2 h at 37 °C. After the incubation period, 0.7 mL of the sample was transferred into another tube containing 3 mL of 0.5% (*w*/*v*) thiobarbituric acid (made in 5 mM sodium hydroxide). The samples were vortex mixed and then incubated for 30 min at 100 °C. After the incubation period, the samples were incubated for 5 min on ice. The tubes were then centrifuged for 5 min at 10,000× *g* rpm, the supernatant was recovered, and 200 µL was loaded into the wells of a microtitre plate. The samples were read at wavelengths of 532 nm and 600 nm on the spectrophotometer. The hydroxyl radical concentration was calculated using the extinction coefficient of 155 mM.cm^−1^ [43]. Three tomato samples per treatment were randomly selected from the three repetitions.

### 4.7. Statistical Analysis

The data collected were plant height (cm); plant dry weight (g); roots dry weight (g); fresh plant weight (g); roots fresh weight (g); FRAP (µmol AAE/g), TEAC (Umol TE/g), superoxide (nmol/g) and hydroxyl contents (nmol/g); and cell death (A600nm/g). There were no significant differences in the growth results when the three replicates were compared; hence, the growth data of the 15 plants (5 from each repetition) of each treatment were pooled. The data were analysed using one-way ANOVA. The number of leaves was analysed using the Kruskal–Wallis test, and the means were separated using the Mann–Whitney test. The level of significance was fixed at *p* < 0.05. The analyses were performed using the statistical software TIBCO Statistica^®^ 13.3.0 Dell Inc., Palo Alto, CA, USA.

## 5. Conclusions

In conclusion, *B. bassiana* inoculum enhanced the growth of tomatoes infected with FOL, improved the antioxidant capacity of plants in the *B. bassiana* treatment, and induced higher oxidative stress levels (ROS contents) in both *F. oxysporum*-infected and-uninfected tomatoes. This study revealed a strong positive association between hydroxyl radical content and plant growth. Future studies involving nutrient uptake, growth hormones, generation mechanisms of hydroxyl radical species, and enzymes in an endophytic fungi–plant–pathogen relationship are needed to better understand the mechanisms through which endophytic fungi enhance plant growth and confer protection to *F. oxysporum*-infected tomatoes. 

## Figures and Tables

**Table 1:** The effect of *B. bassiana* on the growth of tomato plants (n = 60) that were drenched with FOL conidial suspension.

Treatments	Plant Height (cm)	Number of Leaves
T1	36.45 ± 0.45 b	12.27 ± 0.59 ab
T2	30.87 ± 0.96 c	10.80 ± 0.88 c
T3	37.80 ± 0.63 ab	12.87 ± 0.41 b
T4	39.27 ± 0.80 a	14.27 ± 0.4 a

Means with the same lowercase letters in the column indicates means ± SE are not significantly different using the Tukey HSD test (Plant height) and Mann–Whitney test (Number of leaves) at a *p* = 0.05 level of significance. T1, Control; T2, FOL; T3, *B. bassiana*; T4, *B. bassiana* and FOL.

**Table 2:** Effect on the endophytic activity of *B. bassiana* on the fresh weight and dry weight of roots and aerial parts.

Treatments	Aerial Fresh Weight (g)	Roots Fresh Weight (g)	Aerial Dry Weight (g)	Roots Dry Weight (g)
T1	35.68 ± 1.08 b	13.49 ± 0.87 b	3.91 ± 0.17 b	3.71 ± 0.33 b
T2	24.89 ± 1.27 c	9.09 ± 0.71 c	2.59 ± 0.20 c	3.51 ± 0.35 b
T3	54.45 ± 2.06 a	14.55 ± 0.72 ab	5.04 ± 0.30 a	4.21 ± 0.27 ab
T4	57.76 ± 2.47 a	17.38 ± 1.14 a	5.57 ± 0.23 a	5.20 ± 0.35 a

Means with the same lowercase letters in the column indicate that means ± SE are not significantly different using the Tukey HSD (fresh and dry weights) or Mann–Whitney (number of leaves) tests at *p* = 0.05. T1, Control; T2, FOL; T3, *B. bassiana*; T4, *B. bassiana* and FOL.

**Table 3:** Effect of fungal inoculation on antioxidants capacity.

Treatments	FRAP (µmol AAE/g)	TEAC (µmol TE/g)
T1	432.74 ± 25.89 a	696.78 ± 38.38 ab
T2	392.00 ± 22.88 ab	638.25 ± 44.37 ab
T3	418.26 ± 20.19 ab	713.18 ± 37.94 a
T4	346.67 ± 10.99 b	571.35 ± 22.16 b

Means with the same lowercase letters in the column indicates means ± SE are not significantly different using the Tukey HSD test at *p* = 0.05 level of significance. T1, Control; T2, FOL; T3, *B. bassiana*; T4, *B. bassiana* and FOL.

**Table 4:** Effects of endophytic *B. bassiana* on cell death following exposure to pathogenic FOL.

Treatments	Cell Death (A600 nm/g)
T1	0.021 ± 0.001 a
T2	0.025 ± 0.001 a
T3	0.021 ± 0.003 a
T4	0.022 ± 0.001 a

Means with the same lowercase letters in the column indicate that means ± SE are not significantly different using the Tukey HSD test at *p* = 0.05. T1, Control; T2, FOL; T3, *B. bassiana*; T4, *B. bassiana* and FOL.

**Table 5:** Effects of endophytic *B. bassiana* on superoxide (mean ± SE nmol/g) content in tomato leaves following exposure to pathogenic FOL.

Treatments	Superoxide (nmol/g)
T1	45.95 ± 6.66 c
T2	42.28 ± 3.33 d
T3	52.50 ± 5.02 b
T4	61.46 ± 1.96 a

Means with the same lowercase letters in the column indicates means ± SE are not significantly different using the Tukey HSD test at *p* = 0.05 level of significance. T1, Control; T2, FOL; T3, *B. bassiana*; T4, *B. bassiana* and FOL.

**Table 6:** Effects of endophytic *B. bassiana* on Hydroxyl radical (mean ± SE nmol/g) content in tomato leaves following exposure to pathogenic FOL.

Treatments	Hydroxyl Radical (nmol/g)
T1	111.18 ± 5.70 ab
T2	94.28 ± 6.91 b
T3	112.12 ± 4.46 ab
T4	119.29 ± 2.96 a

Means with the same lowercase letters in the column indicate that means ± SE are not significantly different using the Tukey HSD test at *p* = 0.05. T1, Control; T2, FOL; T3, *B. bassiana*; T4, *B. bassiana* and FOL.

## Data Availability

Not applicable.

## References

[1] Lyons R., Stiller J., Powell J., Rusu A., Manners J.M., Kazan K. (2015). *Fusarium oxysporum* triggers tissue-specific transcriptional reprogramming in *Arabidopsis thaliana*. PLoS ONE.

[2] de Lamo F.J., Takken F.L. (2020). Biocontrol by *Fusarium oxysporum* using endophyte-mediated resistance. Front. Plant Sci..

[3] Srinivas C., Devi D.N., Murthy K.N., Mohan C.D., Lakshmeesha T.R., Singh B., Kalagatur N.K., Niranjana S.R., Hashem A., Alqarawi A.A. (2019). *Fusarium oxysporum* f. sp. lycopersici causal agent of vascular wilt disease of tomato: Biology to diversity—A review. Saudi J. Biol. Sci..

[4] Joshi R. (2018). A review of *Fusarium oxysporum* on its plant interaction and industrial use. J. Med. Plants Stud..

[5] Jaber L.R., Ownley B.H. (2018). Can we use entomopathogenic fungi as endophytes for dual biological control of insect pests and plant pathogens?. Biol. Control.

[6] De Silva N.I., Brooks S., Lumyong S., Hyde K.D. (2019). Use of endophytes as biocontrol agents. Fungal Biol. Rev..

[7] Card S., Johnson L., Teasdale S., Caradus J. (2016). Deciphering endophyte behaviour: The link between endophyte biology and efficacious biological control agents. FEMS Microbiol. Ecol..

[8] Rodriguez R., Redman R. (2008). More than 400 million years of evolution and some plants still can’t make it on their own: Plant stress tolerance via fungal symbiosis. J. Exp. Bot..

[9] Gao F.K., Dai C.C., Liu X.Z. (2010). Mechanisms of fungal endophytes in plant protection against pathogens. Afr. J. Microbiol. Res..

[10] Bolívar-Anillo H.J., Garrido C., Collado I.G. (2020). Endophytic microorganisms for biocontrol of the phytopathogenic fungus *Botrytis cinerea*. Phytochem. Rev..

[11] Fadiji A.E., Babalola O.O. (2020). Elucidating mechanisms of endophytes used in plant protection and other bioactivities with multifunctional prospects. Front. Bioeng. Biotechnol..

[12] Prihantini A.I., Tachibana S. (2017). Antioxidant compounds produced by *Pseudocercospora* sp. ESL 02, an endophytic fungus isolated from *Elaeocarpus sylvestris*. Asian Pac. J. Trop. Biomed..

[13] Caruso G., Abdelhamid M.T., Kalisz A., Sekara A. (2020). Linking endophytic fungi to medicinal plants therapeutic activity. A case study on Asteraceae. Agriculture.

[14] Zabalgogeazcoa I. (2008). Fungal endophytes and their interaction with plant pathogens: A review. Span. J. Agric. Res..

[15] Busby P.E., Ridout M., Newcombe G. (2016). Fungal endophytes: Modifiers of plant disease. Plant Mol. Biol..

[16] Bacon C.W., White J.F. (2016). Functions, mechanisms and regulation of endophytic and epiphytic microbial communities of plants. Symbiosis.

[17] White J.F., Torres M.S. (2010). Is plant endophyte-mediated defensive mutualism the result of oxidative stress protection?. Physiol. Plant..

[18] Mascarin G.M., Jaronski S.T. (2016). The production and uses of *Beauveria bassiana* as a microbial insecticide. World J. Microbiol. Biotechnol..

[19] Bamisile B.S., Dash C.K., Akutse K.S., Keppanan R., Wang L. (2018). Fungal endophytes: Beyond herbivore management. Front. Microbiol..

[20] Moloinyane S., Nchu F. (2019). The effects of endophytic *Beauveria bassiana* inoculation on infestation level of *Planococcus ficus*, growth and volatile constituents of potted greenhouse grapevine (*Vitis vinifera* L.). Toxins.

[21] Macuphe N., Oguntibeju O.O., Nchu F. (2021). Evaluating the Endophytic Activities of *Beauveria bassiana* on the Physiology, Growth, and Antioxidant Activities of Extracts of Lettuce (*Lactuca sativa* L.). Plants.

[22] Di X., Takken F.L., Tintor N. (2016). How phytohormones shape interactions between plants and the soil-borne fungus *Fusarium oxysporum*. Front. Plant Sci..

[23] Rojas E.C., Jensen B., Jørgensen H.J., Latz M.A., Esteban P., Ding Y., Collinge D.B. (2020). Selection of fungal endophytes with biocontrol potential against Fusarium head blight in wheat. Biol. Control.

[24] Combès A., Ndoye I., Bance C., Bruzaud J., Djediat C., Dupont J., Nay B., Prado S. (2012). Chemical communication between the endophytic fungus *Paraconiothyrium variabile* and the phytopathogen *Fusarium oxysporum*. PLoS ONE.

[25] Hassan S.E. (2017). Plant growth-promoting activities for bacterial and fungal endophytes isolated from medicinal plant of *Teucrium polium* L.. J. Adv. Res..

[26] Baron N.C., Rigobelo E.C. (2021). Endophytic fungi: A tool for plant growth promotion and sustainable agriculture. Mycology.

[27] Huang H., Ullah F., Zhou D.-X., Yi M., Zhao Y. (2019). Mechanisms of ROS Regulation of Plant Development and Stress Responses. Front. Plant Sci..

[28] Anjum S.A., Ashraf U., Zohaib A., Tanveer M., Naeem M., Ali I., Nazir U. (2017). Growth and developmental responses of crop plants under drought stress: A review. Zemdirb. Agric..

[29] Huang W.Y., Cai Y.Z., Hyde K.D., Corke H., Sun M. (2007). Endophytic fungi from *Nerium oleander* L. (*Apocynaceae*): Main constituents and antioxidant activity. World J. Microbiol. Biotechnol..

[30] Singh Y., Nair A.M., Verma P.K. (2021). Surviving the odds: From perception to survival of fungal phytopathogens under host-generated oxidative burst. Plant Commun..

[31] Fones H., Preston G.M. (2012). Preston. Reactive oxygen and oxidative stress tolerance in plant pathogenic Pseudomonas. FEMS Microbiol. Lett..

[32] Hernández-Chávez M.J., Pérez-García L.A., Niño-Vega G.A., Mora-Montes H.M. (2017). Fungal strategies to evade the host immune recognition. J. Fungus..

[33] Latifian M., Rad B., Amani M. (2014). Mass production of entomopathogenic fungi *Metarhizium anisopliae* by using agricultural products based on liquid-solid diphasic method for date palm pest control. Int. J. Farming Allied Sci..

[34] Rhoda I., Akinpelu E.A., Etsassala N.G., Nchu F. Evaluating Resistance of Five Local Heirloom Tomato Cultivars to the Phytopathogen *Fusarium oxysporum*. Proceedings of the 18th South Africa International Conference on Agricultural, Chemical, Biological & Environmental Sciences (ACBES-20).

[35] Johnson E.A. (1946). An improved slide culture technique for the study and identification of pathogenic fungi. J. Bacteriol..

[36] Raja H.A., Miller A.N., Pearce C.J., Oberlies N.H. (2017). Fungal identification using molecular tools: A primer for the natural products research community. Nat. Prod. J..

[37] Karlsson I., Edel-Hermann V., Gautheron N., Durling M.B., Kolseth A.K., Steinberg C., Persson P., Friberg H. (2016). Genus-specific primers for study of Fusarium communities in field samples. Appl. Environ. Microbiol..

[38] Cherry J.M., Hong E.L., Amundsen C., Balakrishnan R., Binkley G., Chan E.T., Christie K.R., Costanzo M.C., Dwight S.S., Engel S.R. (2012). Saccharomyces Genome Database: The genomics resource of budding yeast. Nucleic Acids Res..

[39] Rehner S.A., Minnis A.M., Sung G.H., Luangsa-ard J.J., Devotto L., Humber R.A. (2011). Phylogenetic systematic of the anamorphic entomophathogenic Beauveria. Mycologia.

[40] Norjmaa U., Nasamdulam D., Enkhjargal B., Banzragch D. (2019). Morphological and molecular identification of Beauveria bassiana from agricultural soils. Mong. J. Agric. Sci..

[41] Benzie I., Strain J. (1996). The Ferric Reducing Ability of Plasma (FRAP) as a Measure of “Antioxidant Power: The FRAP Assay”. Anal. Biochem..

[42] Miller N.J., Rice-Evans C., Davies M.J., Gopinathan V., Milner A. (1993). A novel method for measuring antioxidant capacity and its application to monitoring the antioxidant status in premature neonates. Clin. Sci..

[43] Gokul A., Roode E., Klein A., Keyster M. (2016). Exogenous 3, 3′-diindolylmethane increases *Brassica napus* L. seedling shoot growth through modulation of superoxide and hydrogen peroxide content. J. Plant Physiol..

[44] Halliwell B., Gutteridge J.M.C. (1984). Oxygen toxicity, oxygen radicals, transition metals and disease. Biochem. J..

